# Influence of coffee brewing methods on the chromatographic and spectroscopic profiles, antioxidant and sensory properties

**DOI:** 10.1038/s41598-021-01001-2

**Published:** 2021-11-01

**Authors:** Natalia Stanek, Magdalena Zarębska, Łukasz Biłos, Krzysztof Barabosz, Ewa Nowakowska-Bogdan, Izabela Semeniuk, Justyna Błaszkiewicz, Renata Kulesza, Rafał Matejuk, Krzysztof Szkutnik

**Affiliations:** 1 Łukasiewicz Research Network- Institute of Heavy Organic Synthesis “Blachownia”, Energetyków 9, 47-225 Kędzierzyn-Koźle, Poland; 2 Faculty of Production Engineering and Logistics, Opole University of Technology, Prószkowska 76, 45-758 Opole, Poland; 3 Hard Beans Coffee Roasters, Armii Krajowej 35, 45-071 Opole, Poland

**Keywords:** Chemistry, Engineering

## Abstract

Cold brewing coffee has gained increasing popularity as a novel brewing method. A completely different flavour profile during cold brewing extraction (smooth and mild) is a result of the low-energy process, prolonged water-grind contact times and long preparation time. The aim of our research was to compare coffee drinks obtained with an innovative device for a faster, dynamic cold coffee extraction process (Hardtank) to drinks prepared traditionally in 24 h and hot brewed drinks. This study investigated the differences in chemical composition (volatile, non-volatile and lipid compounds), sensory properties and antioxidant capacity of coffee drinks from various extraction processes carried out at variable brewing temperatures, times and percolation modes. The results showed that the new cold maceration technique using coffee bed percolation (Hardtank) improved the quality of cold coffee drinks, making them similar in taste to hot coffee drinks. Among the studied extractions, the combination of a lower temperature (19.3 °C) and percolation process appeared to be the ideal setting for the most efficient extraction of compounds such as chlorogenic acids, gallic acid, caffeine, trigonelline, 5-(hydroxymethyl)furfural and lipids and consequently for their intake. In addition, FTIR spectra indicated an even 4 times greater quantity of lipids in Hardtank drinks than in classic cold brew and up to 5 times more lipids than in hot brew coffee, which contribute to the formation of the aroma and flavour. The decreased extraction time and use of coffee bed percolation could be beneficial for the quality and taste of cold brew products.

## Introduction

The recently observed trend dominating the functional beverage market is the production of natural drinks containing plant ingredients extracted from parts such as roots, flowers, fruits, leaves and seeds. This tendency depends on sociodemographic and sociocultural differences in consumer perceptions and the acceptance of functional products^1^. The inspiration from nature in creating beverages with benefits for the human body can be summarized by the name “functional beverages” or even “bionic drinks”, which can be defined by rapid, low-temperature extraction technology. The benefits of boosting energy and fat burning and supporting the immune system^2–4^ are constantly increasing the popularity of functional beverages (and bionic drinks) worldwide. In particular, coffee, as well as tea and herbs, has become one of the most popular beverages. Statistics indicate that approximately 2.25 billion cups of coffee are consumed worldwide every day^5^. The cause of this phenomenon not only the flavour, smell and well-known stimulating properties of coffee but also its good influence on health. Coffee extraction is the final step in coffee production and has a great influence on the final form and properties of the beverage. Generally, coffee brewing techniques can be divided into high- and low-temperature processes^6–8^. The most popular methods of hot coffee brewing include the Turkish technique, espresso, filtered (V60, Aeropress, French Press) and simple infusion method^8,9^. Turkish coffee is prepared by boiling the coffee in water, while a simple infusion is based on combining the ground grains with hot water (85–96 °C) and macerating for approximately 3–5 min^6,8^. Espresso coffee is one of the most appreciated infusions by consumers. During preparation, the ground coffee is briefly pressurized with hot water using a percolator to yield a small cup of concentrated coffee^9–11^. Of the various methods that use filters, the most well-known approach is V60. In the V60 method, ground coffee is brewed using 96–98 °C water in a cone-shaped brewing device. This coffee maker consists of three parts: a cone-shaped upper dripper, a paper filter, and a glass vessel. Water is poured into the V60 to create a small crater in the middle of the ground coffee^12,13^. Coffee brewing techniques that do not use high temperatures in the extraction process include cold drips, immersion or a cold French press^14,15^. The share of this product is growing on the global market owing to the undoubted benefits of the cold brew method, such as boosting metabolism, lifting mood and lowering risk of heart diseases, type 2 diabetes, Parkinson’s and Alzheimer’s diseases, in addition to being easier on the stomach than hot coffee. According to the newest report of Grand View Research, Inc., the size of the cold brew coffee global market will grow to 1.63 billion USD by 2025^16^. Despite the many advantages, the low temperature causes a slowed extraction process, which results in a longer steep time, even up to 8–24 h^17,18^. Therefore, it seems necessary to look for alternative methods for the cold extraction process, which could possibly significantly reduce the extraction time. Recently, an innovative, fast and dynamic process performed with the Hardtank device has become an alternative to the traditional 24-h cold maceration of coffee. A device in which the maceration and percolation process of the coffee bed is simultaneously carried out in a properly selected hydrodynamic system allows reducing the extraction time to 30–50 min to obtain optimal output. The coffee particle size degree has great importance in the process, as it depresses the formation of the coffee bed in the filter basket. During the maceration process, the basket with the central mandrel is immersed in water. The mandrel has openings through which water circulates—it is a closed circuit between the container and the coffee bed, subsequently the water containing substances leached out from the bed flows out through the mesh, on which the coffee filter is naturally formed. The rapid cold extraction process enables adaptation of the pressure parameters, which has a significant impact on the sensory quality of the obtained product. This approach is in contrast to the traditional 24-h cold brew maceration where coffee particles are steeped in cold water in a sealed container without any additional agitation or coffee brewed using a pour over set. The rapid cold extraction process enables to adapt the pressure parameters, which has a significant impact on the sensory quality of the obtained product.

Therefore, the aim of this research was to evaluate the effect of three coffee extraction methods, namely, filter hot brew V60 (P), innovative percolated cold brew with the Hardtank (HT) and traditional 24-h cold brew (CB), including the time and temperature of extraction as well as percolation process, on the compounds content (non-volatile, volatile and lipid profile), antioxidant capacity (DPPH and ABTS assays) and sensory profile of beverages (aroma, flavour, aftertaste, acidity, body, balance and overall impression). The differences between the three tested extraction processes carried out at variable brewing temperatures, times and percolation modes were tracked using Arabica beans with different geographic origins (El Salvador, Guatemala, Bolivia and Brazil) and harvesting methods (washed, natural and pulped natural).

## Results and discussion

### HPLC analysis

This section aims to separate, identify and quantify important coffee extract substances, especially three groups. The first group is polyphenolic acids and their derivatives, namely, chlorogenic acids such as 3-O-caffeoylquinic acid (3-CQA), 4-O-caffeoylquinic acid (4-CQA), 5-O-caffeoylquinic acid (5-CQA) and gallic acid. The second group is alkaloids, of which caffeine and trigonelline are the key components, and the third group is represented by the major furfural 5-(hydroxymethyl)furfural (5-HMF).

The levels of the identified compounds are shown in Table 1. The contents of analysed compounds differ in cold brew (CB), Hardtank (HT) and hot brew (P) extracts and depend on the number of factors, such as post-harvest techniques (washed, natural or pulped natural), extraction time (3.5 min, 30 min or 24 h) and brewing temperature (96 °C for hot brew, 19.3 °C for cold brew) or percolation (used in the Hardtank device). The highest content of chlorogenic acids was obtained in the Hardtank process for Bolivian (Bow) and Guatemalan (Gw) coffees, while the lowest was observed in coffee from El Salvador (Sw) and Brazil (Bpn) for both the CB and P processes. The HT process turned out to be more efficient than the traditional CB approach, and in four of the six analysed coffees, it was even more efficient than the hot brew process. In both cases, the elution of CQA isomers was approximately 20% higher in the HT process. Even if cold water brewing extracts some compounds less efficiently than hot^15,17^ simultaneous percolation in selected hydrodynamic systems used in the HT process proved to be more effective. Regarding caffeine, the highest levels were recorded for both washed (Gw, Sw) and natural (Bn, Spn) coffees, while the lowest levels were recorded for BOw coffee, both from the CB and P processes. According to Lane et al.^19^, traditional cold brew preparation methods produce quantities of caffeine similar to that in hot brews, which agrees with our results. Furthermore, the percolated cold brew process released 18% and 12% more caffeine than that by hot and cold extraction, respectively. The content of trigonelline was the highest for Gw, Sw and Bn coffees in the HT process and the lowest in BOw coffee from both the CB and P processes. In both cases, the HT process yield a trigonelline content approximately 13% higher than that with the other processes. The water temperature and pressure affected the caffeine and trigonelline contents. Caprioli et al.^9^ analysed both Robusta and Arabica coffee samples prepared in two espresso machines using different pressures (7, 9, 11 bar) and temperatures (88, 92, 98 °C) to brew coffee. The caffeine content was higher in Arabica bean infusions prepared at a higher temperature and lower pressure (92–98 °C at a pressure of 7 bar), but higher Robusta coffee prepared at lower temperature and higher pressure (88–92 °C at a pressure of 9 bar). In the case of trigonelline, the extraction yield increased as the temperature increased from 88 to 92 °C, while at constant temperature, the increased pressure resulted in lower trigonelline levels. Cordoba et al.^20^ reported that coffee beverages brewed with the cold drip method displayed higher caffeine, trigonelline, and 4- and 5-caffeoylquinic acid (CQA) contents than hot brew (French press), which is in agreement with our results. In all types of analysed extractions, Sw and Spn coffees were richer in 5-HMF than the other coffees. However, also for 5-HMF, the HT process washed out 16% more than that in the CB process and 10% more than that in the P process. The same trend was observed for gallic acid, in which the highest content was recorded in Gw coffee, where HT maceration was approximately 12% more efficient. In our experiment, we noticed that the coffee prepared with the percolated cold extraction method (Hardtank) contained significantly more (p < 0.05) chlorogenic acids, caffeine, trigonelline and 5-(hydroxymethyl)furfural than the conventional cold brew coffee. The greatest quantities of 3-O-caffeoylquinic acid and caffeine were detected in Hardtank coffees, which were in the ranges of 711–897 mg/100 g and 643–795 mg/100 g of coffee, respectively. An exception was a sample from Brazil processed pulped naturally, in which no significant differences between the content of non-volatile compounds leached using the three tested extraction processes were observed.

**Table 1:** The level of identified chemical compounds (mg/100 g) in coffee brews.

[mg/100 g]		Gw	Sw	Bpn	Bn	Spn	BOw
5-CQA	CB	260 ± 2^c^	227 ± 1^b^	236 ± 1^a^	261 ± 17^b^	262 ± 19^b^	280 ± 3^b^
	HT	288 ± 1^a^	268 ± 2^a^	235 ± 1^a^	283 ± 2^a^	265 ± 4^a^	294 ± 4^a^
	P	279 ± 2^b^	229 ± 5^b^	234 ± 7^a^	234 ± 15^c^	239 ± 5^c^	263 ± 13^c^
3-CQA	CB	784 ± 2^c^	678 ± 4^c^	710 ± 4^a^	800 ± 52^a^	763 ± 19^a^	866 ± 8^a^
	HT	858 ± 7^b^	806 ± 6^a^	711 ± 3^a^	858 ± 4^a^	812 ± 6^a^	897 ± 11^a^
	P	871 ± 10^a^	695 ± 14^b^	727 ± 22^a^	733 ± 49^b^	735 ± 10^b^	815 ± 39^b^
4-CQA	CB	321 ± 6^b^	281 ± 14^b^	284 ± 2^a^	331 ± 22^b^	306 ± 8^a^	356 ± 4^a^
	HT	357 ± 3^a^	324 ± 2^a^	284 ± 2^a^	359 ± 2^a^	333 ± 3^a^	370 ± 5^a^
	P	353 ± 6^a^	278 ± 6^b^	286 ± 8^a^	303 ± 20^c^	299 ± 5^b^	335 ± 16^b^
Caffeine	CB	711 ± 9^c^	668 ± 17^c^	732 ± 14^a^	705 ± 44^b^	743 ± 20^b^	605 ± 13^b^
	HT	790 ± 3^a^	785 ± 10^a^	730 ± 12^a^	770 ± 4^a^	795 ± 20^a^	643 ± 15^a^
	P	770 ± 3^b^	705 ± 17^b^	740 ± 15^a^	689 ± 40^b^	764 ± 10^b^	601 ± 24^b^
Trigonelline	CB	618 ± 6^c^	597 ± 4^b^	574 ± 7^a^	566 ± 34^b^	556 ± 6^b^	520 ± 4^b^
	HT	687 ± 3^a^	667 ± 6^a^	573 ± 6^a^	634 ± 4^a^	579 ± 20^a^	566 ± 5^a^
	P	645 ± 3^b^	603 ± 10^b^	539 ± 11^b^	559 ± 29^b^	564 ± 18^b^	523 ± 18^b^
5-HMF	CB	58 ± 4^c^	63 ± 0^b^	49 ± 0^b^	50 ± 3^b^	61 ± 1^c^	54 ± 0^c^
	HT	64 ± 2^a^	72 ± 0^a^	50 ± 0^a^	57 ± 0^a^	68 ± 1^a^	60 ± 1^a^
	P	60 ± 0^b^	69 ± 1^a^	50 ± 1^a^	52 ± 3^b^	66 ± 3^b^	56 ± 2^b^
Gallic acid	CB	45 ± 2^b^	25 ± 0^b^	23 ± 1^c^	30 ± 2^b^	30 ± 1^c^	32 ± 0^c^
	HT	49 ± 0^a^	28 ± 1^a^	24 ± 0^b^	34 ± 0^a^	33 ± 2^a^	36 ± 0^a^
	P	44 ± 0^b^	27 ± 0^a^	26 ± 0^a^	30 ± 1^b^	31 ± 1^b^	33 ± 0^b^

The values represent average ± standard deviation, n = 6.

*3-CQA* 3-O-caffeoylquinic acid, *4-CQA* 4-O-caffeoylquinic acid, *5-CQA* 5-O-caffeoylquinic acid, *5-HMF* 5-(Hydroxymethyl)furfural, *CB* cold brew, *HT* Hardtank, *P* brewed coffee, *Gw* Guatemala washed, *Sw* El Salvador washed, *Bpn* Brazil pulped natural, *Bn* Brazil natural, *Spn* El Salvador pulped natural, *Bow* Bolivia washed.

The superscripts a, b, c denote significant (p < 0.05) differences between Hardtank, traditional cold and hot brewing method within the same coffee sample.

### GC–MS analysis

GC–MS was used to investigate how Coffee varieties differ from each other in the content and intensity of aroma notes. Profiles of volatile compounds consist of dozens of volatile chemicals. The differences in their content result from the applied production chain, which includes: geographical origin of the grains, climate in the place of cultivation, type of grain used, harvesting method, processing, i.e., roasting and grinding, as well as storage conditions and brewing method (time–temperature profile)^21,22^. This paper tries to detail the aroma differences resulting from the brewing methods in coffees of different origins.

From several dozen identified compounds, 12 were selected that had the most intense signal and assigned positive or negative flavour attributes^23^. These compounds belong to 9 functional groups, i.e., aldehydes (2-methylbutanal, furfural, 5-methyl-2-furfural), ketones (2,3-butanedione, 2,3-pentanedione), alcohols (2-furanmethanol), acetates (furfuryl acetate), furans (2-methylfuran), pyrazines (2,5-dimethylpyrazine, 2-ethyl-3-methylpyrazine, 3-ethyl-2,5-dimethylpyrazine), phenolic compounds (4-vinylguaiacol) and sulfur-containing compounds (dimethyldisulfide). Based on the literature, it is possible to assign aromatic notes^21–23^ as well as the attractiveness of the aroma, which can be positive or negative^23^ (Table 2). Qualitative analysis was carried out based on the standard mixture, and quantitative analysis was based on an internal standard.

**Table 2:** Key volatile compounds detected in the coffee brews grouped by chemical class with compared odours and comparison of compounds content in [μg/mL] found in coffee brews via HS-SPME/GC–MS.

[μg/mL]		Gw	Sw	Bpn	Bn	Spn	BOw
**Aldehydes**							
3-Methylbutanal Malt, fermented Negative^23^	CB	0.329 ± 0.026^b^	0.221 ± 0.005^c^	0.432 ± 0.028^a^	0.159 ± 0.000^a^	0.346 ± 0.003^b^	0.562 ± 0.001^a^
	HT	0.421 ± 0.013^a^	0.304 ± 0.001^a^	0.158 ± 0.063^b^	0.160 ± 0.001^a^	0.413 ± 0.006^a^	0.477 ± 0.002^b^
	P	0.159 ± 0.003^c^	0.028 ± 0.004^b^	0.265 ± 0.004^b^	0.106 ± 0.013^a^	0.217 ± 0.010^c^	0.091 ± 0.000^c^
Furfural Sweet, woody, almond, baked bread fragrant Positive^22^	CB	2.484 ± 0.129^a^	0.615 ± 0.005^b^	0.548 ± 0.066^a^	0.377 ± 0.003^a^	0.544 ± 0.008^a^	1.002 ± 0.002^a^
	HT	2.454 ± 0.037^a^	0.679 ± 0.003^a^	0.346 ± 0.020^b^	0.393 ± 0.001^a^	0.580 ± 0.005^a^	0.743 ± 0.005^b^
	P	1.837 ± 0.003^b^	0.677 ± 0.000^a^	0.567 ± 0.001^a^	0.370 ± 0.045^a^	0.554 ± 0.028^a^	0.638 ± 0.001^c^
5-Methyl-2-furfural Spice, caramel, maple Positive^21,22^	CB	0.379 ± 0.041^a,b^	0.277 ± 0.002^b^	0.317 ± 0.018^a^	0.186 ± 0.012^a^	0.358 ± 0.003^a^	0.490 ± 0.002^a^
	HT	0.406 ± 0.005^a^	0.305 ± 0.004^a^	0.242 ± 0.004^b^	0.209 0.001^a^	0.378 ± 0.000^a^	0.416 ± 0.000^b^
	P	0.298 ± 0.011^b^	0.274 ± 0.001^b^	0.309 ± 0.005^a^	0.183 ± 0.014^a^	0.359 ± 0.009^a^	0.344 ± 0.001^c^
**Ketones**							
2,3-Butanedione Buttery Positive^22^	CB	0.073 ± 0.008^a,b^	0.011 ± 0.000^b^	0.044 ± 0.012^a^	0.047 ± 0.000^a^	0.059 ± 0.002^b^	0.062 ± 0.000^a^
	HT	0.085 ± 0.003^a^	0.008 ± 0.000^c^	0.017 ± 0.005^a^	0.041 ± 0.001^a^	0.089 ± 0.004^a^	0.032 ± 0.001^c^
	P	0.054 ± 0.000^b^	0.025 ± 0.001^a^	0.039 ± 0.000^a^	0.028 ± 0.003^b^	0.054 ± 0.002^a^	0.052 ± 0.000^b^
2,3-Pentanedione Buttery, caramel like, fruity Positive^23^	CB	0.373 ± 0.045^b^	0.236 ± 0.004^a^	0.264 ± 0.005^a^	0.175 ± 0.002^a^	0.245 ± 0.000^b^	0.386 ± 0.001^a^
	HT	0.516 ± 0.002^a^	0.208 ± 0.003^b^	0.128 ± 0.034^b^	0.182 ± 0.000^a^	0.291 ± 0.004^a^	0.316 ± 0.000^b^
	P	0.312 ± 0.001^b^	0.186 ± 0.000^c^	0.227 ± 0.004^a^	0.151 ± 0.018^b^	0.193 ± 0.009^c^	0.224 ± 0.000^c^
**Alcohols**							
2-Furanmethanol Sweet, smoky, caramel, coffee Positive	CB	2.130 ± 0.001^a^	1.109 ± 0.012^b^	1.162 ± 0.099^a^	0.822 ± 0.003^a^	1.166 ± 0.009^a^	1.980 ± 0.011^a^
	HT	2.259 ± 0.116^a^	1.168 ± 0.000^a^	1.284 ± 0.013^a^	0.811 ± 0.002^a^	1.104 ± 0.017^a^	1.501 ± 0.002^b^
	P	2.211 ± 0.004^a^	1.133 ± 0.005^b^	1.327 ± 0.006^a^	0.836 ± 0.100^a^	1.116 ± 0.052^a^	1.394 ± 0.003^c^
**Acetates**							
2-Furanmethanol acetate Ethereal-floral, herbal-spicy Positive^21,25^	CB	0.391 ± 0.069^a,b^	0.348 ± 0.120^a^	0.529 ± 0.037^a,b^	0.295 ± 0.000^a^	0.473 ± 0.007^b^	0.582 ± 0.007^a^
	HT	0.485 ± 0.008^a^	0.472 ± 0.000^a^	0.603 ± 0.006^a^	0.325 ± 0.005^a^	0.531 ± 0.005^a^	0.476 ± 0.000^b^
	P	0.289 ± 0.001^b^	0.342 ± 0.002^a^	0.438 ± 0.015^b^	0.198 ± 0.014^a^	0.390 ± 0.014^c^	0.294 ± 0.000^c^
**Furans**							
2-Methylfuran Pungent, fruity Negative^21^	CB	0.057 ± 0.002^b^	0.011 ± 0.000^b^	0.013 ± 0.001^a^	0.014 ± 0.000^a^	0.023 ± 0.001^b^	0.024 ± 0.000^b^
	HT	0.072 ± 0.001^a^	0.022 ± 0.000^a^	0.014 ± 0.001^a^	0.018 ± 0.000^a^	0.030 ± 0.002^a^	0.030 ± 0.000^a^
	P	0.026 ± 0.000^c^	0.004 ± 0.000^c^	0.012 ± 0.000^a^	0.009 ± 0.001^b^	0.010 ± 0.000^c^	0.009 ± 0.000^c^
**Pyrazines**							
2,5-Dimethylpyrazine Nutty, roasted, grassy Positive^21^	CB	0.334 ± 0.022^b^	0.100 ± 0.000^c^	0.168 ± 0.014^a^	0.109 ± 0.000^a^	0.193 ± 0.004^a,b^	0.253 ± 0.001^a^
	HT	0.395 ± 0.004^a^	0.134 ± 0.000^a^	0.159 ± 0.005^a^	0.113 ± 0.000^a^	0.203 ± 0.000^a^	0.198 ± 0.001^b^
	P	0.322 ± 0.001^b^	0.116 ± 0.000^b^	0.168 ± 0.000^a^	0.100 ± 0.012^a^	0.174 ± 0.008^b^	0.171 ± 0.000^c^
2-Ethyl-3-methylpyrazine Nutty, peanut, musty Negative^24^	CB	0.020 ± 0.002^a.b^	0.026 ± 0.000^c^	0.008 ± 0.004^a^	0.038 ± 0.000^a^	0.013 ± 0.010^a^	0.018 ± 0.000^a^
	HT	0.025 ± 0.000^a^	0.037 ± 0.000^a^	0.014 ± 0.000^a^	0.045 ± 0.000^a^	0.019 ± 0.000^a^	0.010 ± 0.000^c^
	P	0.018 ± 0.000^b^	0.034 ± 0.000^b^	0.013 ± 0.000^a^	0.036 ± 0.004^a^	0.016 ± 0.001^a^	0.013 ± 0.000^b^
**Phenolic compounds**							
4-Vinylguaiacol Woody, dry, roasted, clove-like Positive^22,24^	CB	0.004 ± 0.001^a^	0.002 ± 0.000^a^	0.001 ± 0.000^b^	0.002 ± 0.000^a^	0.001 ± 0.000^b^	0.003 ± 0.000^a^
	HT	0.005 ± 0.000^a^	0.002 ± 0.000^a^	0.001 ± 0.000^b^	0.002 ± 0.000^a^	0.002 ± 0.000^a^	0.002 ± 0.000^b^
	P	0.004 ± 0.000^a^	0.002 ± 0.000^a^	0.002 ± 0.000^a^	0.001 ± 0.000^b^	0.001 ± 0.000^b^	0.002 ± 0.000^b^
**Sulfur compounds**							
Dimethyldisulfide Cabbage-like Negative^25^	CB	0.009 ± 0.000^a^	0.001 ± 0.000^a^	0.002 ± 0.000^a^	0.001 ± 0.000^a^	0.003 ± 0.000^a^	0.004 ± 0.000^a^
	HT	0.010 ± 0.000^a^	0.001 ± 0.000^a^	0.003 ± 0.001^a^	0.001 ± 0.000^a^	0.003 ± 0.000^a^	0.004 ± 0.000^a^
	P	0.006 ± 0.000^b^	0.001 ± 0.000^a^	0.002 ± 0.000^a^	0.001 ± 0.000^a^	0.002 ± 0.000^b^	0.003 ± 0.000^b^

Mean value ± standard deviation, n = 6. The superscripts a, b, c denote significant (p < 0.05) differences between Hardtank, traditional cold and hot brewing method within the same coffee sample. *CB* cold brew, *HT* Hardtank, *P* Hot brew coffee, *Gw* Guatemala washed, *Sw* El Salvador washed, *Bpn* Brazil pulped natural, *Bn* Brazil natural, *Spn* El Salvador pulped natural, *Bow* Bolivia washed.

As seen from the data presented in Table 2, the highest values are achieved by furan derivatives containing methyl, acetate, aldehyde, aldehyde and methyl groups as well as –OH groups. Furfural and 2-furanmethanol in particular stand out in terms of concentrations above those of other compounds. Both of these compounds reached the highest values in Guatemalan coffee. Although their presence may raise concerns, in 2016, the International Agency for Research on Cancer (IARC) finally concluded that the potential carcinogenicity due to the presence of furans in coffee qualifies as type 3, “not classifiable as to its carcinogenicity to humans”^24^. These compounds are formed during the thermal degradation of endogenous components in the Maillard reaction, which makes them an indispensable element of grain processing. The aromatic notes assigned to these compounds were classified as positively influencing the aroma. The remaining aldehyde-3-methylbutanal is a product of Strecker degradation. The greatest amount of this compound with a malt, fermented note was registered in Bolivian coffee obtained by the CB method. Ketones represented by 2,3-butanedione and 2,3-pentanedione were described as compounds with sweet and buttery notes^23^. In the tested coffees, the highest concentration was obtained for the first ketone in Salvadoran (Spn) and Guatemalan coffees, especially with HT, while the second ketone was obtained from Guatemalan beans with HT. The pyrazines that are listed in Table 2, differ significantly in aroma note. 2-Ethyl-3-methylpyrazine, due to its heavy, musty smell, has been classified as aromatic with a negative impact on the composition of beverages. In the tested samples, the content of this negative compound was approximately an order of magnitude lower than that from the literature. Although the literature indicates that those compounds are noticeable at higher temperatures, in our experiment, the highest concentration was found in HT extracts. The highest values of 2,5-dimethylpyrazine was detected in Guatemalan coffee and 2-ethyl-3-methylpyrazine in Brazilian (Bn) and Salvadoran (Sw) brews. Typically, a higher brewing temperature of coffee determines the greater release of volatile compounds from the prepared drink. As shown by the data collected in Table 2, the difference between the hot brew (P) and the Hard tank method is statistically insignificant and in some cases HT has an advantage over the filter hot method (such as with 2,3-pentanedione in Spn coffee).

### FTIR-ATR analysis

**Figure 1:**
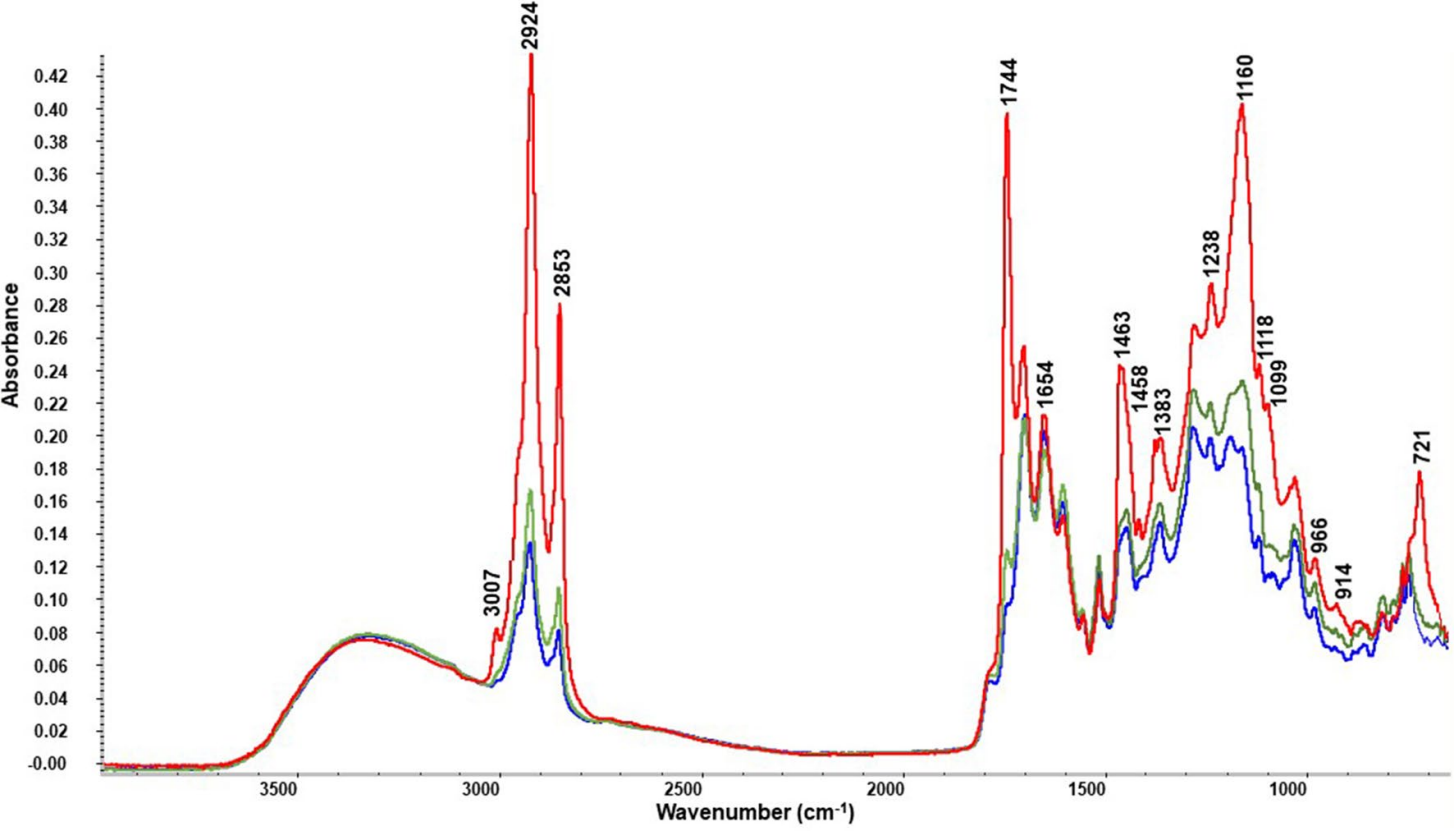
Comparison of FTIR spectra: Cold Brew (blue line), Hot Brew (green line) and Hardtank (red line) for the exemplary Brazil natural (Bn) coffee sample.

The FTIR spectra showed a unique relation of chemical compounds present in hot brew, classic cold brew and Hardtank coffee, indicating that the tested brews are a rich matrix of biomolecules. The analysis of coffee samples was based on the differences in peak intensity and frequency. The representative spectra are shown in Fig. 1. Coffee samples revealed changes in the intensity of some absorption bands, as follows: 1280 and 1605 cm^−1^, associated with chlorogenic acids; 3123, 3011, 1703 and 1653 cm^−1^ with caffeine; 1150 cm^−1^, associated with polysaccharides; and 1600–1500 cm^−1^ as the trigonelline region. Additionally, the most relevant changes were noticed in the lipid regions, as follows: 3007 cm^−1^ (C–H stretching vibrations of cis double bonds (HC=CH)), 2924 and 2853 cm^−1^ (asymmetric and symmetric stretching vibration of C–H bonds in aliphatic CH_2_ groups of the fatty acid backbone), 2956 cm^−1^ (symmetric stretching vibrations of C–H bonds in aliphatic CH_3_ groups), 1744 cm^−1^ (stretching vibrations of ester carbonyl functional groups in triglycerides (O–C=O)), 1463 and 1458 cm^−1^ (bending vibrations of C–H in CH_2_ and CH_3_ aliphatic groups) 1365 cm^−1^ (bending symmetric vibrations of C–H in CH_2_ groups), 1245 and 1160 cm^−1^ (stretching and rocking vibrations of C–O ester groups), 1118 and 1099 cm^−1^ (stretching vibrations of C–O ester groups), 966 cm^−1^ (out-of-plane bending vibrations of trans –HC = CH– groups) 914 cm^−1^ (out-of-plane bending vibrations of cis-HC =CH– groups), and 721 cm^−1^ (aliphatic CH_2_ rocking vibrations). The composition of lipids was identified in the highest quantity in all analysed Hardtank coffee samples (2–4 times more than that in classic cold brew coffee and 2–5 times more than that in hot brew coffee). The lipid fraction represents 7–17% of coffee beans and depends on the botanical origin of coffee (Arabica coffee contains 5% more than Robusta)^25^. Green coffee mainly consists of triacylglycerols, sterols and tocopherols. Additionally, so-called coffee oil contains kaurene esterified diterpenes in proportions of up to 20% of the total lipids. Coffee lipid components are of interest due to their antioxidant, antimicrobial, antiproliferative activity (sterols, tocopherols, free diterpenes, waxes), anti-inflammatory properties and potential anticarcinogenic effects (diterpenes: cafestol and kahweol)^26,27^. Lipids also contribute to the formation of the aroma and flavour of coffee drinks, which is especially important for coffee consumers. The pressure and percolation process of cold brew carries more lipids than traditional brewing methods, so Hardtank coffee should taste better and be favourably accepted by consumers.

### TPC, TFC and antioxidant activity (DPPH and ABTS assays)

Coffee is a unique brew containing a variety of compounds (phenolic and non-phenolic)^28^ with potential radical scavenging ability, and as shown above, their amounts depend on the method of coffee preparation. As there are many origins of coffee beans and brewing methods, it is important to find the optimal method for maximizing antioxidant concentrations. Therefore, one of the goals of the work was to assess the effect of coffee brew preparation (traditional 24-h cold brew, percolated cold brew withHardtank and filter hot brew) on their antioxidant activity.

The data obtained for the total phenolic content (TPC), total flavonoid content (TFC) and all antioxidant assays (DPPH, ABTS assays) for coffee infusions prepared in different brewing processes from beans of various geographical origins are presented in Table 3. Almost all coffee samples extracted by hot coffee brewing showed the highest contents of total phenolics and flavonoids, which ranged from 22.02 to 33.46 mg gallic acid equivalents (GAE) per g coffee and 3.18 to 4.08 mg quercetin equivalents (QE) per g of coffee, respectively. The only exception was the coffee samples from Brazil (Bn), where the amounts of total phenolic compounds of hot and classic cold coffee brews were at the same level. Compared to that in hot coffee, the concentration of phenolic and flavonoid compounds in classic cold brew (24 h) was significantly lower (p < 0.05). The antioxidant activity obtained with DPPH and ABTS methods confirmed the same trend; the hot brewing process involved a higher energy system than the classic cold brewing process, which resulted in a faster rate of soluble antioxidant compound extraction. The highest differences in TPC and TFC values, significantly standing out among all other results, were obtained for coffee samples from Guatemala Gw (23% for TPC, 21% for TFC) and El Salvador Spn (21% for TPC and TFC). Overall, the results of this study for hot and cold brew coffee agree well with the general knowledge regarding the TPC and antioxidant activity of coffee. For example, Fibrianto et al.^29^ also observed that the hot brewing method tended to result in a higher TPC content (20.77 mg GAE/g) than the cold brewing method (19.13 mg GAE/g). Similarly, Rao et al.^17^ indicated that the antioxidant activity was higher in hot brew coffee (18.34–20.72 mmol Trolox/L coffee). High temperature appears to be a key factor affecting the extraction yield of antioxidant compounds. On the other hand, a high water temperature causes phenolic acids and oils (compounds that give coffee a distinct flavour) to degrade and oxidize more quickly, making coffee beverages more acidic and bitter^15^. Therefore, in recent years, alternative cold coffee brewing methods have been explored focusing on reducing the long extraction time required by the traditional method^30^ while making the extraction yield similar to that of hot brewing methods. Comparing the values of TPC, TFC and antioxidant capacities for Hardtank coffees with those for classic cold brews, Hardtank as a cold prepared drink differs significantly and stands out with higher values (p < 0.05). The antioxidant characteristics of Hardtank coffee were slightly better for Sw, Bn, BOw and slightly worst for Gw, Bpn and Spn coffee samples than those of hot brew coffee, but the differences were not very high (2–12% for DPPH and 1–13% for ABTS).

**Table 3:** Comparison of coffee infusions prepared in different brewing processes of the effects on their bioactive and antioxidant properties.

[mg/g]		Gw	Sw	Bpn	Bn	Spn	BOw
TPC [mgGAE/g]	CB	25.92 ± 1.87^b^	21.50 ± 0.68^b^	20.23 ± 3.27^b^	23.46 ± 0.44^b^	18.69 ± 1.29^b^	23.77 ± 0.61^b^
	HT	30.94 ± 0.95^a^	22.51 ± 0.35^a,b^	19.86 ± 2.63^b^	25.14 ± 0.52^a^	24.37 ± 2.00^a^	25.22 ± 1.51^a,b^
	P	33.46 ± 1.10^a^	23.04 ± 0.89^a^	22.02 ± 2.49^a^	23.43 ± 0.62^b^	23.56 ± 1.13^a^	26.90 ± 1.31^a^
TFC [mg/QE/g]	CB	3.07 ± 0.13^b^	3.04 ± 0.05^b^	2.87 ± 0.04^b^	3.11 ± 0.22^a^	3.24 ± 0.11^b^	3.18 ± 0.12^a^
	HT	3.22 ± 0.18^b^	3.57 ± 0.09^a^	3.16 ± 0.17^a^	3.23 ± 0.11^a^	3.30 ± 0.07^b^	3.11 ± 0.12^a^
	P	3.67 ± 0.06^a^	3.70 ± 0.11^a^	3.18 ± 0.07^a^	3.44 ± 0.19^a^	4.08 ± 0.18^a^	3.39 ± 0.23^a^
DPPH [mgTE/g]	CB	33.73 ± 0.71^b^	39.80 ± 0.33^b^	46.53 ± 0.56^a^	37.86 ± 0.53^b^	34.41 ± 0.50^b^	54.68 ± 0.49^a^
	HT	52.55 ± 1.22^a^	45.15 ± 0.76^a^	45.72 ± 1.29^a^	50.41 ± 1.56^a^	52.86 ± 1.48^a^	55.52 ± 1.44^a^
	P	51.14 ± 1.56^a^	39.75 ± 1.68^b^	49.12 ± 2.17^a^	49.17 ± 3.02^a^	53.75 ± 2.86^a^	55.02 ± 2.77^a^
ABTS [mgTE/g]	CB	34.99 ± 2.53^b^	27.63 ± 2.88^b^	33.36 ± 0.86^b^	39.08 ± 1.20^a^	50.71 ± 1.47^b^	42.87 ± 1.02^a,b^
	HT	42.72 ± 0.82^a^	33.04 ± 2.20^a^	31.80 ± 0.84^b^	41.82 ± 0.82^a^	57.45 ± 1.00^a^	44.84 ± 0.69^a^
	P	45.53 ± 0.34^a^	32.61 ± 1.14^a^	35.89 ± 1.19^a^	40.03 ± 1.74^a^	54.72 ± 2.13^a,b^	39.07 ± 1.47^b^

Mean value ± standard deviation, n = 6.The superscripts a, b, c denote significant (p < 0.05) differences between Hardtank, traditional cold and hot brewing method within the same coffee sample *CB* cold brew, *HT* Hardtank, *P* Hot brew coffee, *Gw* Guatemala washed, *Sw* El Salvador washed, *Bpn* Brazil pulped natural, *Bn* Brazil natural, *Spn* El Salvador pulped natural, *Bow* Bolivia washed.

### Sensory analysis

**Figure 2:**
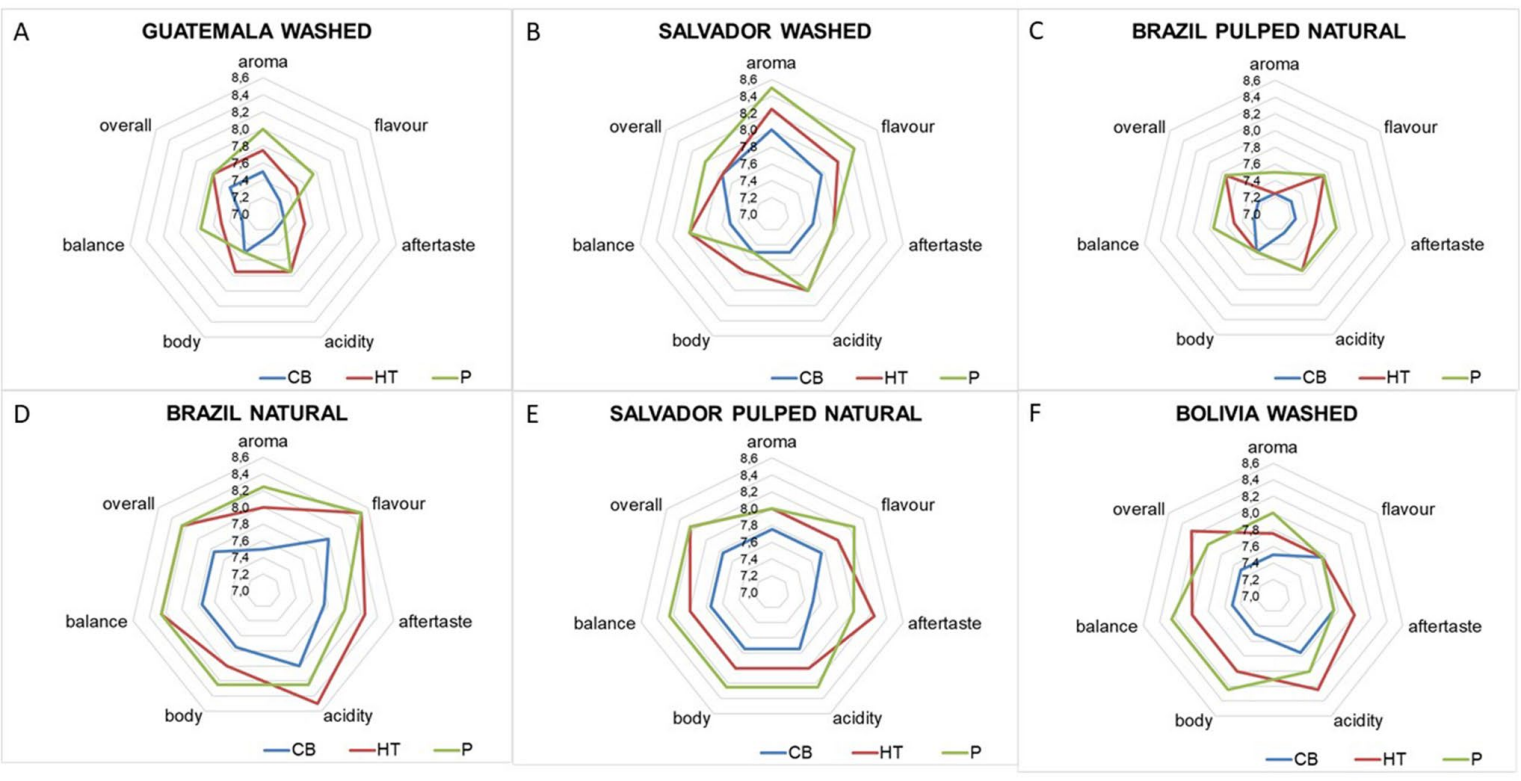
Sensory profiles of coffee samples: (**A**) Guatemala washed, (**B**) El Salvador washed, (**C**) Brazil pulped natural, (**D**) Brazil natural, (**E**) El Salvador pulped natural, (**F**) Bolivia washed. Mean values n = 10.

**Figure 3:**
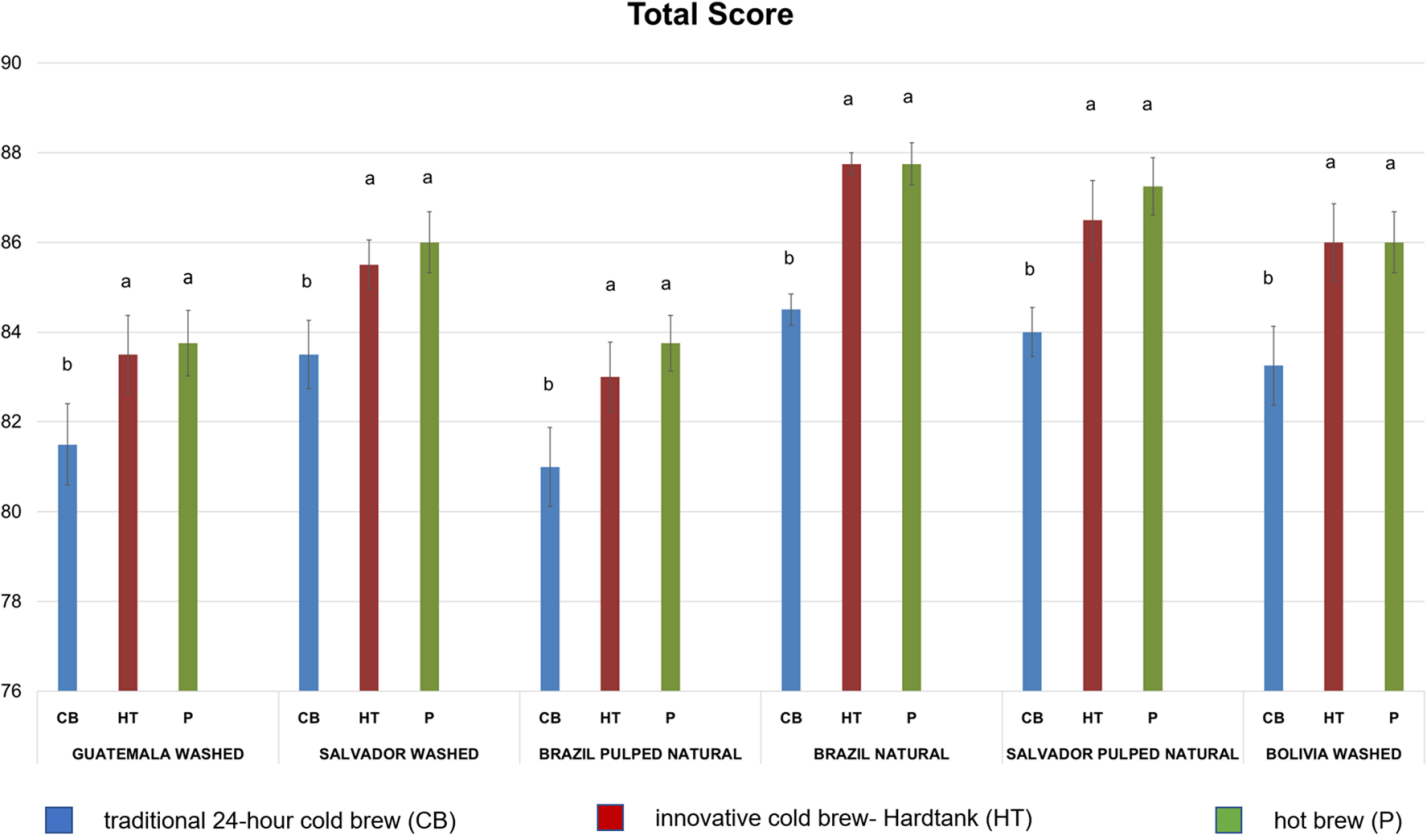
Comparison of total score of coffee samples. The superscripts a, b denote significant (p < 0.05) differences between Hardtank, traditional cold and hot brewing method within the same coffee sample. Mean value ± standard deviation, n = 10.

The sensory analysis carried out according to the SCAA cupping protocol allowed evaluating the descriptor characteristics for the three analysed brewing methods: traditional cold brew, percolated cold brew (Hardtank) and hot brew^31^. Among coffees prepared using the cold extraction method, those from the CB process were characterized by low acidity and short and sharp aftertaste and muted and oxidized flavours in contrast to coffees prepared using the innovative cold extraction method, which were determined by bright acidity, complex flavours, and clean and long-lasting aftertaste. Hot brewing coffees were characterized mostly by intense flavours, sweet but sharp aftertaste and balanced acidity. The results from the sensory analysis for the cold and hot brew coffees are shown in Figs. 2 and 3. The highest total scores among all coffees were observed for hot brews and brews obtained using HT technology (Fig. 3). Together with beverages brewed using Hario V60 Switch (hot brew), coffees obtained through the Hardtank method clearly stand out from coffees prepared in a traditional way (p < 0.05). Regarding global flavour characteristics (Fig. 2), the obtained results indicate higher aroma, flavour, aftertaste, acidity, balance and overall scores of hot brews and Hardtank brews than those for all cold brew coffee treatments. An exception was the body descriptor, in which no significant differences were observed between hot and cold brews for washed El Salvador, washed Guatemala and Brazil processed pulped natural samples. Some sensory characteristics in cold brew coffees were less intense than those in hot beverages, which is in accordance with Cordobo et al.^7^. Cold brew coffees were found to be less acidic than their hot brew counterparts, which has been corroborated by other authors^10^. However, in terms of acidity, extracts from the HT process were more similar or equal to the hot brews. Among all analysed coffees, Brazil natural (Bn) represented the highest attributes while Brazil pulped natural (Bpn) turned out to be less rich. The results of this work revealed that traditional cold brew coffees present flavour profiles with lower aroma, flavour, aftertaste, acidity, balance and overall intensity than those of their hot brew counterparts. In contrast, percolated cold brews (Hardtank) presented very similar values when compared to their hot counterparts. Many discriminators had higher scores when maceration and percolation processes of the coffee bed were employed.

### Statistical analysis

To illustrate the differentiation of the analysed brews (6 types of coffees, 3 extraction methods), in terms of non-volatile compounds, sensory attributes, antioxidant capacity, TPC and TFC, chemometric analysis such as PCA and AHC was applied as a model of 18 cases and 20 variables.

**Figure 4:**
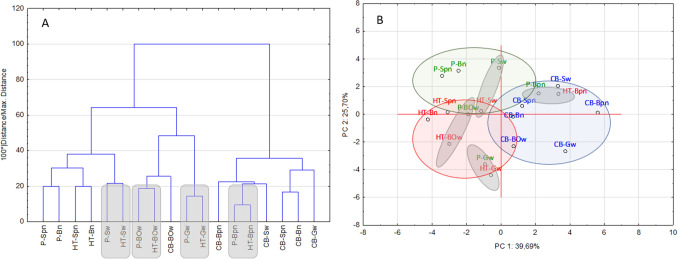
Chemical, antioxidant and sensory discriminants for coffee brews. (**A**) Agglomerative hierarchical cluster analysis- dendrogram. (**B**) Principal component analysis (Statistica, ver. 10, StatSoft).

On the basis of the presented dendrogram (Fig. 4A) it can be concluded that hot brews from the washed El Salvador, Bolivia and Guatemala coffee as well as pulped natural Brazil coffee, show similarity to their Hardtank counterparts (grey marking). The cluster analysis technique proved to be helpful in differentiating the traditional 24-h cold brew from hot and percolated cold brews, which show similarity.

Next, PCA was performed to explain the differences between the analysed coffee brews (Fig. 4B). These results are shown in the system of the two first principal components: PC1 and PC2. Two main factors with eigenvalues higher than one explain over 65% of the data variance while the first distinguished factor (PC1) explains nearly 40% of the variance, and its eigenvalue of 7.9 indicates that it contains information originally explained by 8 variables used to describe the research object. PC1 is the component along which the sensory and antioxidant activity discriminants change, while PC2 is negatively correlated with phenolic compounds (CQAs, gallic acid and TPC) and explains more than 25% of the model variation. The factorial arrangement allowed grouping the coffee brews depending on the extraction method into 3 main groups: green circle, hot brews (the location of which depends on high sensory notes); blue circle, cold brews (where low sensory scores and phenolic compounds are observed); and red circle, percolated cold brews (whose position is determined by high levels of both sensory notes and phenolic compounds). Additionally, PCA enabled observing the similarities between washed processed Bolivian, Guatemalan and Salvadoran coffees as well as natural pulped Brazilian coffees prepared as both hot and Hardtank coffees (grey circles), which confirms the AHC observations.

## Conclusions

The obtained results will help to understand the impact of the coffee preparation method (filter hot brew and cold brews: traditional 24-h and innovative 30-min, percolated version (Hardtank)) on the potential health benefits of the obtained drinks as indicated by the chemical composition, sensory traits and antioxidant properties. Among the analysed coffees, the highest contents of the determined compounds were obtained in Hardtank extracts (p < 0.05). By HPLC analysis, the HT extraction process turned out to be more efficient in terms of leaching chlorogenic acids, caffeine, trigonelline, gallic acid and 5-HMF in relation to both other extraction methods, while for the traditional 24-h cold brewing approach, these differences were the most significant. Among the quantified compounds, 3-O-caffeoylquinic acid and caffeine were detected in the greatest amounts in coffee samples in the ranges of 711–897 mg/100 g and 643–795 mg/100 g of coffee, respectively. The FTIR spectra showed the most relevant changes in the lipid regions in coffee samples. Lipids contribute to the formation of the aromas and flavours of coffees, so their amount in beverages is very important. The highest quantity of lipids was identified in the Hardtank coffee samples, even 4 times more than in classic cold brew coffee and up to 5 times more than that in hot brew coffee. This finding suggests that Hardtank brews can provide more taste sensation. In addition, the sensory test indicated that hot brew and percolated cold brew were preferred over cold-extracted coffee. Many descriptors (aroma, flavour, aftertaste, acidity, balance, overall) had higher scores when the hot brew and Hardtank methods were employed. The Hardtank method also led to extracts with a higher antioxidant activity and phenolic compound content than that from the traditional 24-h cold brewing approach. Based on the laboratory evaluations, it can be concluded that reducing the extraction time to 30 min and using a percolation process allows obtaining beverages of significantly better quality than those with the traditional method.

## Materials and methods

### Chemicals and reagents

Analytical standards of chlorogenic acid (3-CQA, CAS: 327-97-9), neochlorogenic acid (5-CQA, CAS: 906-33-2), caffeine (CAS: 58-08-2), trigonelline (CAS: 535-83-1), gallic acid (CAS: 149-91-7), 5-hydroxymethyl furfural (5-HMF, CAS: 67-47-0), quercetin (CAS: 117-39-5), (±)-6-hydroxy-2,5,7,8-tetramethylchromane-2-carboxylic acid (Trolox, CAS: 53188-07-1), 3-methylbutanal (CAS: 590-86-3), furfural (CAS: 98-01-1), 5-methyl-2-furfural (CAS: 620-02-0), 2,3-butanedione (CAS: 431-03-8), 2,3-pentanedione (CAS: 600-14-6), 2-furanmethanol (CAS: 98-00-0), furfuryl acetate (CAS: 623-17-6), 2-methylfuran (CAS: 534-22-5), 2,5-dimethylpyrazine (CAS: 123-32-0), 2-ethyl-3-methylpyrazine (CAS: 15707-23-0), 3-ethyl-2,5-dimethylpyrazine (CAS: 13360-65-1), 4-vinylguaiacol (CAS: 7786-61-0), dimethyldisulfide (CAS: 624-92-0) and toluene (CAS: 108-88-3) were purchased from Sigma, Fluka, Chromadex, POL-AURA and POCH. All standards used were of analytical grade (≥ 99% purity). The mobile phase in HPLC was prepared by diluting formic acid (Fisher Scientific, LC–MS purity, CAS: 64-18-6) with ultrapure water (Direct-Q system, resistivity below 18 MΩ cm) to a concentration of 0.1% (w/w) and adjusting the pH was to 2.4 and methanol (POCH, HPLC grade, CAS: 67-56-1). To quantify the antioxidant capacity of the coffee brews, DPPH (2,2-diphenyl-1-picrylhydrazyl, Sigma-Aldrich, CAS: 1898-66-4) and ABTS (2,2′-azino-bis(3-ethylbenzothiazoline-6-sulfonic acid) diammonium salt, POL-AURA, CAS: 30931-67-0) was used as the sources of free radicals. All other chemicals and solvents were of analytical grade.

### Coffee samples

The coffee cherries were harvested from mature coffee trees (*Coffea arabica* L.) in El Salvador, Guatemala, Bolivia and Brazil. Then, the obtained raw coffee beans were processed using three different types of coffee processing methods.

#### Washed (w)

Coffee cherries are placed into water to sort out the ripe and unripe cherries. Ripe fruits will sink to the bottom, and unripe fruits will float to the top. The next step is to remove the skin and pulp using a de-pulping machine. The seeds moved through a channel into a large water tank where the ale is left for approximately 18–48 h for fermentation. However, this duration can fluctuate depending on different factors (variety, weather, elevation, region). Once fermentation is performed, the seeds are removed from the water, placed on patios or raised beds and left to dry. The seeds are dried to a moisture content ranging from 10 to 12%.

#### Natural (n)

Coffee cherries are not de-pulped until they are fully dry (approximately 11% moisture content). Microorganisms present both in the fruit and in the environment will perform fermentation in the coffee until it is dried. On average, this process takes from 15 to 25 days.

#### Pulped natural (pn)

Coffee cherries are placed into a de-pulping machine to remove the skin. The mucilage-covered seeds are moved to a drying surface, typically on patios, where they will ferment for 15–20 days on average until they reach a proper moisture content (approximately 11%).

After farm processing, coffee beans were packed and shipped to Europe (Poland) and stored in fully controlled conditions at 13 °C/50% relative humidity until the roasting process began. The roasting process of each coffee sample was performed using a fully controllable drum roaster (Diedrich CR 35 Zenyth II) 48 h before the extraction process started. The characteristics of the studied samples are shown in Table 4.

**Table 4:** The characteristic of studied coffee samples.

Sample label	Geographical origin	Region	Altitude [masl]	Processing method	Fermentation	Drying
Gw	Guatemala	Huehuetenango	1900	Washed	24 h	14–21 days on patios
Sw	El Salvador	Usulutan	1600	Washed	24–36 h	21–28 days on raised beds
Bpn	Brazil	Campo das vertentes	900	Pulped naturally	15–20 days on raised beds	
Bn	Brazil	Campo das vertentes	1000–1200	Naturally	10–15 days on patios	
Spn	El Salvador	Usulutan	1500–1600	Pulped naturally	15–20 days on raised beds	
BOw	Bolivia	Samaipata	1500–1700	Washed	48 h	60–75 h machine dryer

*A statement on guidelines* as experimental research and field studies on plants (either cultivated or wild), is comply with relevant institutional, national, and international guidelines and legislation. Studies are comply with local and national regulations—formal ethical approval is not required.

### Coffee brewing methods

The coffees were prepared by a method that ensures the repeatability of the roasting, grinding and extraction process.

Cold brew (CB) coffees were prepared using 300 g of ground coffee (Mahlkoenig VTA 6S grinder, grind setting: 5) immersed in 5000 mL of RO AKVO 120 ppm quality water at 19.3 °C. The coffee was macerated in a cooling chamber at 10 °C for 24 h and then stirred for 30 s before being filtered on a 0.28 mm paper filter Cafec T-90 Medium Dark Roast.

Hardtank (HT) bionic coffees were prepared using 600 g of ground coffee (Mahlkoenig VTA 6S, grind setting: 5) and 10,000 mL of water of the same quality and temperature as that in the CB method. The maceration and percolation process was carried out for 30 min.

HarioV60 Switch (P) coffees were brewed using 21 g of ground coffee (Mahlkoenig EK43, grind setting: 8) and 350 mL of 96 °C water of the same quality as that in previous methods. Filtration was carried out using a 0.28 mm paper filter Cafec T-90 Medium Dark Roast. The total brewing time was 3.5 min.

All described operations for each method were carried out to obtain constant TDS (total dissolved solids) values measured using an Atago RX-5000i refractometer. For each sample, the electrical conductivity (EC) was determined using an ALMEMO 710 V7 conductivity meter (AHLBORN). The EC values of coffee infusions ranged from 2.51 to 2.77 mS/cm for the cold brew method, 2.60 to 2.94 mS/cm for Hardtank and from 2.47 to 2.65 mS/cm for the hot brew method (Hario V60).

### HPLC analysis

Chromatographic analysis was performed on a Dionex UltiMate 3000 chromatograph equipped with an automatic pump, injector, autosampler, column compartment, and UV–Vis detector with photodiode array (PDA) technology. Instrument control, data acquisition and processing were managed by Chromeleon 6.8 software. Compounds were separated with an isocratic reverse-phase system. A 100 × 4.6 mm Kinetex XB C18 chromatographic column with iso-butyl side chains and TMS endcapping stationary phase (3.5 μm, Phenomenex) and a guard column with similar composition were employed. The column compartment was monitored at 30 °C during all chromatographic runs. The mobile phase consisted of formic acid solution in water (0.1%, w/w) as solvent A and methanol as solvent B. The elution conditions were: 85% A and 15% B (0–12 min). During all analysis times, the flow rate of the mobile phase remained at 1.0 mL/min and the injection volume was 1 µL. The wavelengths scanned were 325 nm for 3-CQA, 4-CQA, and 5-CQA and 272 nm for trigonelline, gallic acid, 5-HMF and caffeine. Identification of individual compounds in the coffee brew samples was achieved by comparing their retention times and UV–Vis spectral characteristics with those of the original standards. With regard to the quantitative analysis, calibration curves were obtained by injection of known concentrations of different standard materials of analysed substances. The content of the compounds was calculated based on 5–8 point calibration curve, and each point was injected in duplicate. The calibration curves were linear with correlation coefficient (r) values greater than 0.99 in the following working ranges expressed in mg/100 g and regression line equations: 5-CQA and 4-CQA, 2–20, y = 2.2463x − 0.0364; 3-CQA, 18–190, y = 1.9225x − 1.4616; trigonelline, 13–68, y = 0.6772x − 0.3425; gallic acid, 0.8–9.2, y = 0.9285x − 0.0047; 5-HMF, 0.7–6.0, y = 1.9808x − 0.0337; and caffeine, 23–117, y = 1.6474x − 1.2918. The LODs and LOQs expressed in mg/100 g were: 5-CQA and 4-CQA: 22, 72, respectively; 3-CQA: 288, 960; trigonelline: 31, 102; gallic acid: 36, 120; 5-HMF: 12, 40; and caffeine: 45, 151.

### GC–MS analysis

Volatile compounds were determined by headspace solid phase microextraction gas chromatography mass spectroscopy (HS-SPME/GC–MS) according to^23^ with slight adaptions. First, 3 mL of the beverage was placed in a 20 mL glass vial and sealed with a Teflon cap with a silicone septum. Then, 10 µL of a standard solution of toluene in water (concentration of 0.3 mg/mL) was added to the vial. Adsorption was carried out using fibre (Agilent Technologies SPME Fibre DVB/C-WR/PDMS). The analysis was performed on Agilent Technologies 7890A system with an MSD detector 7000 GC/MS Triple Quad and computer program “MassHunter”. Each sample was equilibrated for 30 min at 80 °C. Next, volatile compounds were desorbed by placing the fibre into a GC–MS system (injector temperature of 240 °C) for 3 min. Compound separation was conducted on a Phenomenex ZB-5MSplus column (30 m × 250 μm i.d. 0.25 µm film) using helium as carrier gas (flow 1 mL/min). The oven temperature program started with a 3-min isotherm of 50 °C, and then, the temperature was increased by 10 °C/min up to 240 °C, which was maintained for 8 min. Each analysis was carried out in duplicate. Mass spectra were acquired in electron impact (EI) mode at 70 eV using an m/z range of 40–500. To tensure fibre purity, before use, it was conditioned as recommended by the manufacturer and tested to evaluate the consistency of its performance versus the reference coffee sample. Compounds were identified based on their retention time (RT) on ZB-5MSplus column, their mass spectra, and the injection of pure standards. The mass spectra were compared with those from the NIST 08 library. The relative concentration of each identified volatile substance was defined by manually integrating its peak area and calculating with respect to the internal standard (toluene) and expressed in μg/mL.

### FTIR-ATR analysis

Spectroscopic data of the beverage extracts were acquired by FTIR spectrometer (Nicolet 6700, Thermo Scientific, USA) equipped with a 60 °C ZnSe ATR accessory (Thermo Scientific, USA). The FTIR-ATR spectra were recorded in the wavelength range of 4000–650 cm^−1^ using 32 scans per sample at a resolution of 4 cm^−1^. For each sample, the absorbance spectrum was collected against a background obtained with a dry and empty ATR cell. Sample preparation: 50 mL of macerate was extracted with 10 mL of diethyl ether in a separating funnel for 2 min. After 15 min, the ether layer was separated, and then, the extraction was repeated with 5 mL of fresh solvent. Next, 2 mL of the ether extract was applied to the ZnSe crystal and allowed to dry. The residual material that remained on the crystal after the solvent evaporated was then characterized by FTIR analysis.

### Total phenolic content

The Folin-Ciocalteu method was used to spectrophotometrically determine total phenolic compounds as described by Singleton^32^. Briefly, 400 µL of prepared solutions (i.e., 20 µL of coffee extracts diluted with distilled water to 1 mL) was mixed with 200 µl of Folin–Ciocalteau reagent (FC) and 600 µL of 20% Na_2_CO_3_ solution. Then, they were diluted to 4 mL and kept in the dark for 120 min. After this time, the absorbance was measured at 765 nm (Spectrophotometer HP-Hewlett Packard, type: 8452A) against the blank. The TPC was expressed as milligrams of gallic acid equivalents (GAE) mg per 1 g of coffee and was determined as the average from three parallel replicates measured in duplicate.

### Total flavonoid content

The total flavonoid content was determined using the colorimetric method of Chang et al.^33^ with some modifications. Briefly, 100 µL of coffee extract was mixed with 900 µL of MeOH, 60 µL of AlCl_3_ solution (10%) and 60 µL of CH_3_COONa (1 N). The mixture was diluted with distilled water to 4 mL and allowed to stand for 30 min. The absorbance was measured at 420 nm against the blank (mixture of water and reagent). The results were expressed as quercetin equivalents (QE) in milligrams per 1 g of coffee, and determined as the average from three parallel replicates measured in duplicate.

### DPPH assay

The antiradical activity of the coffee extract was evaluated by a modified method of Brand-Williams et al.^34^. First, 1 mL of coffee extract (10 µL of coffee brew with 990 µL of MeOH) was mixed with 3 mL of a methanolic solution of DPPH radical (0.1 mM). The control test was performed with methanol lacking the extract. The mixture was mixed and allowed to stand in the dark for 30 min. The absorbance was measured at 517 nm against the blank (methanol). The antiradical activity of the coffee samples was presented as mg Trolox equivalent/g of coffee.

### ABTS assay

The antioxidant activity of coffee samples was determined by the method described by Re et al., (1999)^35^. ABTS^∙+^ was obtained in the reaction of 1 mL of ABTS [0.01 M 2,2-azino-bis (3-ethylbenzothiazoline-6-sulfonic acid) diammonium salt] with 1 mL of potassium persulfate (0.005 M). The mixture was left to stand in the dark for 20 h to reach optimal absorbance at 734 nm. Then 300 μL of each coffee extract (20 μL coffee brew/1 mL of distilled water) was mixed with 3 mL of the ABTS^∙+^ solution, and after 6 min, the absorbance was measured against a blank (water). The antiradical activity of the coffee samples was calculated as mg Trolox equivalent per g of coffee.

### Sensory analysis

The sensory evaluation of coffee samples was performed using scaling method described by SCAA^31^. Five certified judges in two independent blind cuppings evaluated seven sensorial attributes (aroma, flavour, aftertaste, acidity, body, balance and overall impression). Each of these characteristics was rated on a 16-point scale from 6 to 10, and the final score was calculated by summing the individual scores given for each attribute, additionally taking into account clean cup, uniformity and sweetness. The coffee samples were roasted 48 h before the extraction process, after which coffee beverages were poured into standardized cupping vessels and assessed at room temperature.

### Statistical analysis

Classification techniques, such as agglomerative hierarchical cluster analysis (AHC, dendrogram using Ward’s method with Euclidean distance) and principal component analysis (PCA), were used to interpret the obtained results. The mean values were compared by ANOVA and a Tukey HDS post hoc test. The calculations were performed using Statistica ver. 10 (www.statsoft.pl). A correlation matrix was used to find significant correlations between the considered variables. Differences were considered significant when the correlation coefficient was greater than the absolute value of 0.4 and the p value was < 0.05. For each analysed extraction method (cold brew, Hardtank, hot brew), three independent extraction experiments were performed and analysed (HPLC, GC, TPC, TFC, DPPH and ABTS assays) in duplicate (n = 6). For the sensory analysis, the assessment was made by five judges in two independent cuppings (n = 10).

## Coffee brewing methods

CB: Traditional 24-h cold brew method
HT: Innovative, fast and percolated cold extraction method (Hardtank)
P: Filter hot brew method (HarioV60 Switch)

## Coffee sample labels

Gw: Guatemalan washed processed coffee
Sw: Salvadoran washed processed coffee
Bpn: Brazilian pulped natural processed coffee
Bn: Brazilian natural processed coffee
Spn: Salvadoran pulped natural processed coffee
Bow: Bolivian washed processed coffee
